# Arrhythmia-provoking factors and symptoms at the onset of paroxysmal atrial fibrillation: A study based on interviews with 100 patients seeking hospital assistance

**DOI:** 10.1186/1471-2261-4-13

**Published:** 2004-08-03

**Authors:** Anders Hansson, Bjarne Madsen-Härdig, S Bertil Olsson

**Affiliations:** 1 Department of Cardiology, University Hospital, Lund, Sweden

**Keywords:** Paroxysmal atrial arrhythmia, triggering factors, symptoms, alcohol.

## Abstract

**Background:**

Surprisingly little information on symptoms of paroxysmal atrial fibrillation is available in scientific literature. Using questionnaires, we have analyzed the symptoms associated with arrhythmia attacks.

**Methods:**

One hundred randomly-selected patients with idiopathic paroxysmal atrial fibrillation filled in a structured questionnaire.

**Results:**

Psychic stress was the most common factor triggering arrhythmia (54%), followed by physical exertion (42%), tiredness (41%) coffee (25%) and infections (22%). Thirty-four patients cited alcohol, 26 in the form of red wine, 16 as white wine and 26 as spirits. Among these 34, red wine and spirits produced significantly more episodes of arrhythmia than white wine (p = 0.01 and 0.005 respectively).

Symptoms during arrhythmia were palpitations while exerting (88%), reduced physical ability (87%), palpitations at rest (86%), shortage of breath during exertion (70%) and anxiety (59%). Significant differences between sexes were noted regarding swollen legs (women 21%, men 6%, p = 0.027), nausea (women 36%, men 13%, p = 0.012) and anxiety (females 79%, males 51%, p = 0.014).

**Conclusion:**

Psychic stress was the commonest triggering factor in hospitalized patients with paroxysmal atrial fibrillation. Red wine and spirits were more proarrhythmic than white wine. Symptoms in women in connection with attacks of arrhythmia vary somewhat from those in men.

## Background

Atrial fibrillation (AF) is nowadays divided into three different forms; paroxysmal, persistent and permanent [1]. Even if the pathological, electrical and physiological phenomena leading to AF have been described in ever more detail, the mechanisms underlying these changes remain largely unknown. The relative occurrences of paroxysmal atrial fibrillation (PAF) and other forms of this arrhythmia in the population are also not well known. In a material based on hospital observations, 35% of all fibrillation was described as being of paroxysmal type [2]. Despite being common, surprisingly limited information about possible triggering factors and symptoms at the onset of arrhythmia in larger groups of patients is available [3]. Our aim in this investigation was to throw further light on the factors believed by patients to have caused their arrhythmia and on the symptoms experienced at the onset of attacks of AF.

## Methods

A group of one hundred patients suffering ECG-verified PAF and whose symptoms prompted hospital care were asked to fill in a structured questionnaire with 58 questions covering arrhythmia-triggering factors, time at which the attack starts and symptoms during attack. All patients completed the data by personal interviews by one of the authors. The vast majority of all patients were recruited during two periods of totally 14 months. In some questions, extra information could be supplied by the patients in their own words. (The complete questionnaire could be seen in additional file 1: AHansson_enkat.pdf). All patients had earlier had attacks of AF which stopped either spontaneously or following medication. Most of them had earlier been treated in hospital for the arrhythmia. Patients who previously had myocardial infarction, pericarditis, diseases of the thyroid or diabetes were not included since these diseases may be the underlying cause of the arrhythmia [4-10]. Our group of patients thus had idiopathic PAF. Permission for the investigation was obtained from the local Ethical Committee and all patients were informed about the investigation both orally and in writing.

### Statistics

Data were compared using Fisher's Exact Test and Mann Whitney U-test. Values of p < 0.05 were considered significant.

## Results

### Material

**Figure 1:**
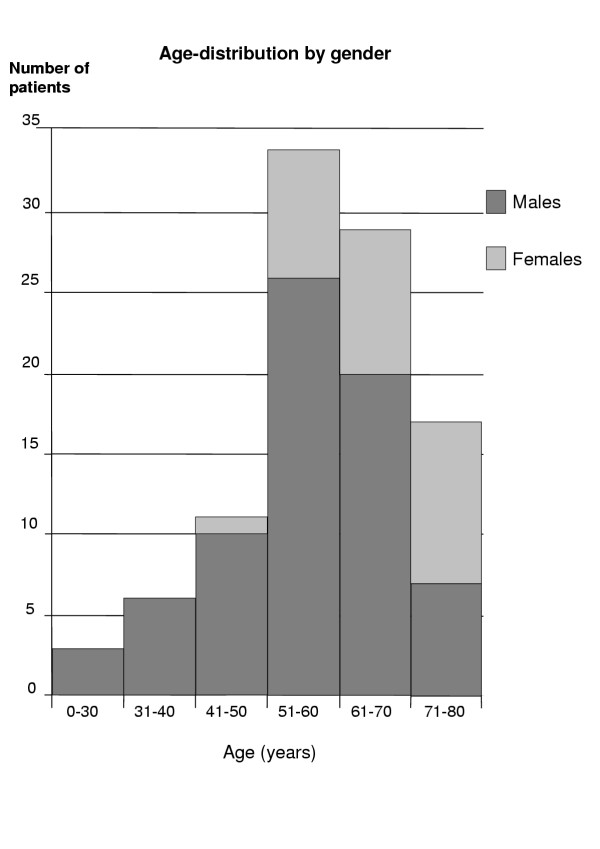
**Age-distribution by sex. **In the group investigated, the median age was 59.9 (22.4–79.2) years median (range). The women were significantly older than the men.

One hundred completed questionnaires were filled in by 72 men and 28 women. Twenty-four of these had on some occasion been treated for hypertension. The median (range) age of the entire group was 59.9 (22.4–79.2) years. Patients with previous histories of hypertension were significantly older than those without 65.0 (46.6–78.6) years as against 58.6 (22.4–79.2) years respectively. The women in the group were significantly older than the men 67.9 (54.1–78.6) years and 58.2 (22.4–79.2) years respectively, p = 0.02 (Fig. 1). Since patients with hypertension differed from the others only as regards age, the ensuing analyses were made without respect to its incidence. Seventy six of all patients were given genuine antiarrhythmic drugs, sotalol being the most common. Twenty four of the patients were not taking any antiarrhythmic pharmacological treatment (Table 1).

**Table 1:** Current pharmacological antiarrhythmic treatment

Sotalol	40
Disopyramid	13
Digoxin	13
Metoprolol	5
Flekainid	5
Propranolol	3
Atenolol	3
Verapamil	3
Bisoprolol	1
Amiodaron	1
No antiarrhythmic medication	24

### Attacks of arrhythmia

**Figure 2:**
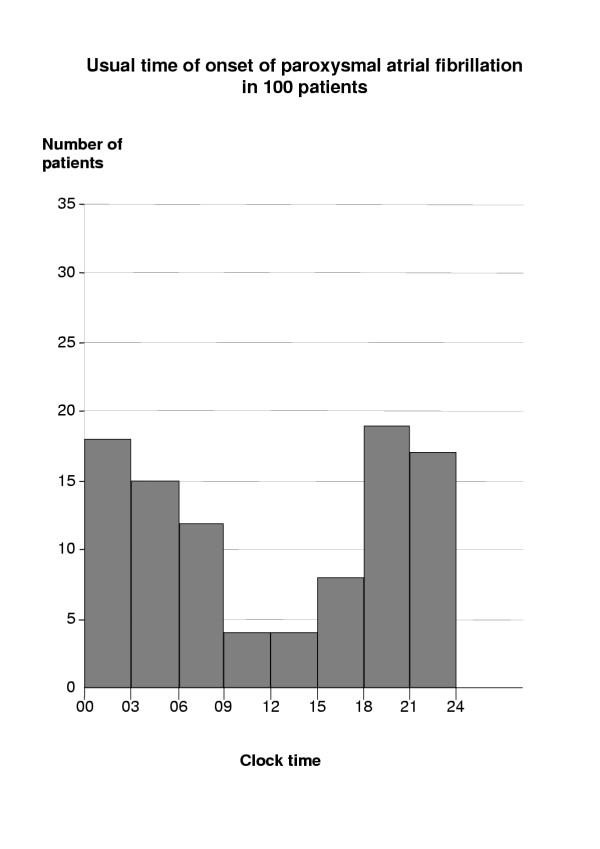
**Time at which palpitations started. **Seventy-two patients thought that arrhythmia always started at the same time of day, typically in the evening or at night. Some, however, gave more than one time-interval.

Seventy-two patients believed that their attacks usually started at about the same time of day, typically starting in the evenings or at night (Fig. 2). The time of onset appeared not to depend on sex.

For most patients (64%), attacks typically lasted less than one day, while a further 17% gave 1–7 days. No attack lasted more than one week.

Thirty-five percent of the entire group woke up with episodes of fibrillation, 34% stated that attacks began with psychic stress, as defined by the patients. Thirty one percent of the episodes started during rest and 22% of the attacks followed physical exertion. Twenty-five percent could not think of any special factor initiating attacks. No differences in the onset of attacks between men and women could be established. Eighty-five percent of the group succeeded in identifying some sort of triggering-factor (Table 2). The most common of these was psychic stress followed by physical exertion, tiredness and infection. As regards foodstuffs, 25% thought that coffee was the triggering-factor. Those who cited sympathetic tone anamnesis with stress as the triggering-factor usually stated that the onset of arrhythmia was in the evening or at night.

**Table 2:** Possible arrhythmiatriggering-factors as identified by patients with paroxysmal atrial fibrillation

**Triggering factor**	**%**
Mental stress	54
Physical effort	42
Tiredness	41
Any alcohol	34
White wine	16
Red Wine	26
Liquor	26
Coffee	25
Infections	22
Cold drinks	8
Large meal	3
Food	18
Onions	5
Nuts	4
Chocolate	3
Ice cream	2
Spiced food	2
Cream	1
Strawberries	1
Fish	1
Sweets	1
Beans	1
Shellfish	1
Garlic	1
No triggering-factor	15

**Figure 3:**
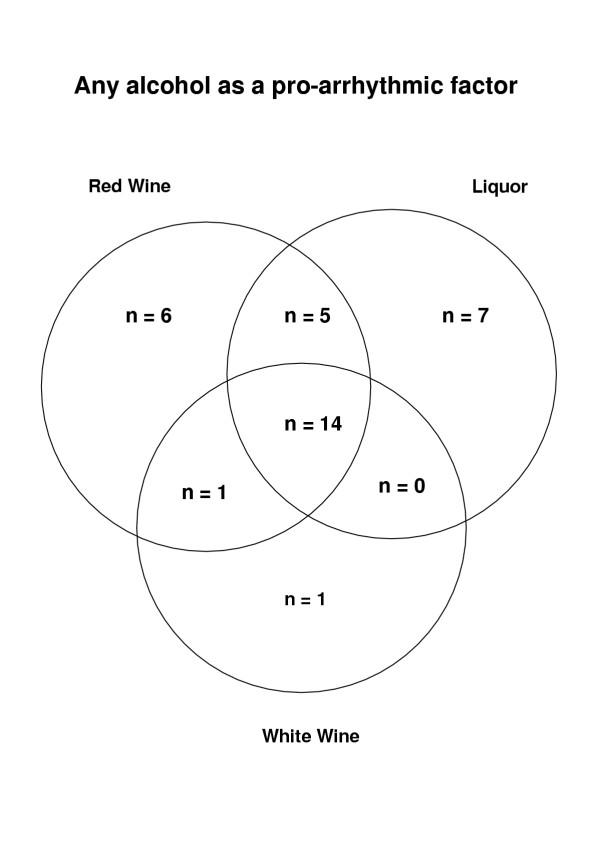
**Any alcohol as a pro-arrhythmic factor. **Thirty-four patients cited various forms of alcohol as a factor triggering arrhythmia. Some patients named more than one kind of alcohol. Twenty-six cited red wine, 26 spirits and 16 white wine. In this group, red wine and spirits caused significantly more episodes of arrhythmia than white wine.

Alcohol was named as a triggering-factor by 34 patients, in 26 cases in the form of red wine, in 26 as spirits and in 16 white wine. Some patients named more than one kind of alcohol as a triggering factor. Two of those who reacted to red wine did not drink spirits and hence could not say whether spirits, too, trigger arrhythmia. In the alcohol-triggered group, red wine and spirits triggered more episodes of arrhythmia than white wine (p = 0.01 and p = 0.05 respectively). There were no significant differences in the arrhythmia-provoking effects of spirits and red wine nor between men and women (Table 2, Fig. 3). Time delay between onset of trigger and subsequent onset of the AF episode was not explored in the questionnaire.

Foodstuffs such as onion, nuts, chocolate and ice-cream were cited as agents producing fibrillation by a few patients (Table 2).

Seventy-four percent considered that the episodes of AF they experienced affected their lifestyles, while 26% thought this was not the case. Among answers in their own words, a common reply was that they did not dare to exercise as much as they would have liked (13 patients). Eight patients did not dare to travel. Sixty-six percent stated that their episodes of AF affected their relatives, while 32% answered this question in the negative. In their own words, they remarked that the main problem was their relatives' uneasiness.

During attacks of AF, the most common symptoms were palpitations in connection with strain, reduced physical performance, palpitations when at rest, breathlessness during exertion and anxiety (Table 3). Females showed significantly higher frequencies of swollen legs (p = 0.02), indisposition (p = 0.012) and anxiety (p = 0.014) than males.

**Table 3:** Symptoms in association with the onset of paroxysmal atrial fibrillation by gender

**Symptoms**	**Total n = 100**	**Males n = 72**	**Female n = 28**	**p-value**
Pre-symptoms	32	20 (28%)	12 (43%)	0.159
Pains in the chest	25	14 (19%)	11 (39%)	0.070
Dizziness	52	33 (46%)	19 (68%)	0.074
Syncope	7	4 (6%)	3 (11%)	0.396
Breathlessness when resting	41	32 (44%)	9 (32%)	0.365
Breathlessness when working	70	53 (74%)	17 (61%)	0.230
Swollen ankles	10	4 (6%)	6 (21%)	0.027
Palpitation at rest	86	62 (86%)	24 (86%)	>0.999
Palpitation at exercise	88	59 (82%)	23 (82%)	>0.999
Nausea	19	9 (13%)	10 (36%)	0.012
Vomiting	2	1 (1%)	1 (4%)	0.484
Abdominal pain	5	2 (3%)	3 (11%)	0.312
Loss of appetite	31	20 (28%)	11 (39%)	0.336
Anxiety	59	37 (51%)	22 (79%)	0.014
Reduced physical capacity	87	62 (86%)	25 (89%)	>0.999
Polyuria*	40/75	25/52 (48%)	15/23 (65%)	0.213

*this question was put to only 75 patients.

## Discussion

Although PAF is one of the most common heart-disturbances causing patients to get in touch with medical care centres, surprisingly little information is available about the factors which trigger it and the symptoms associated with the onset of arrhythmia in larger groups of patients. This study has therefore been undertaken to determine which factors patients consider responsible for triggering arrhythmia and the symptoms that occur in connection with episodes of arrhythmia.

### What provokes arrhythmia?

From observations of heart-rate in sinus rhythm shortly before its onset, a separation of PAF into sympathetically-mediated and vagal forms has been suggested [11]. Earlier studies have shown a degree of daily variation in the onset of AF. Thus, attacks are more common in the morning and at night [12], but higher frequency has also been reported during daytime [13]. A possible explanation is that arrhythmia often starts in younger patients at night and in older ones during the day [14]. A weekly variation has also been reported, with fewer attacks on Saturdays [12]. An annual variation with fewer attacks during the last months of the year has also been reported [12].

In our study, the 72 patients who thought that their attacks of arrhythmia usually occurred at about the same time of day gave this as the evening or at night. Hence a large fraction of those investigated should have vagal PAF since this often starts at night [11]. Despite this, the majority of the patients considered that the triggering-factor was some kind of situation in which increased levels of catecholamines can be discerned. Even those with positive stress-related anamnesis (triggered by physical exertion and psychic stress) had often attacks starting in the evening or at night. However, this need not imply an absolute correlation in time, but rather the probable existence of a certain latent period between stress and the onset of arrhythmia. In earlier studies, it has been proposed that attacks of PAF are often due to variations in the tonus of the autonomic nervous system. Arrhythmia is stimulated particularly when an initial adrenergic increase is followed by an abrupt change to vagal dominance [15].

Alcohol has long been considered to play an etiological role in PAF, a correlation underlined in the expression "holiday heart" [11]. Since temporary enhanced alcohol consumption is frequent, it is difficult to prove a direct correlation [8,16]. Episodes of AF have been triggered by the acute effects of alcohol on atrial refractoriness and conduction, but also by the effects of chronic misuse of alcohol leading to subclinical heart dysfunction [17]. Other mechanisms have also been discussed [18]. Every third patient in our study considered alcohol to be a triggering-factor, but white wine was blamed less than red wine and spirits. Why arrhythmia should be triggered less frequently by white wine than by red wine or spirits remains unclear.

It is now generally accepted that most attacks of PAF are induced by ectopic impulses originating in the pulmonary veins [19,20]. Both automaticity and triggered automaticity in these cells have been demonstrated in experimental conditions [19,20]. The influence of the autonomic nervous system, alcohol and other factors inducing arrhythmia on this mechanism is, however, uncertain.

### Symptoms at the onset of fibrillation

Although a number of patients with PAF are without any symptoms [21], in general patients with this form of arrhythmia show more symptoms than those with permanent AF [22]. Studies with telephone-transmitted ECG have, however, shown a sensitivity of symptomatic registrations of up to 89% with PAF [23]. There is thus good correlation between the symptoms and ECG-verified AF.

The limited amount of literature on the symptomology of PAF includes Quality of Life investigations, Case Reports and quantification of a few symptoms [24]. Investigations based on "Quality of Life" forms have earlier shown that patients with PAF have lower scores for physical function, emotional function, vitality and general health [24]. The symptoms commonly reported include palpitations, giddiness, dyspnoea, tachycardia, perspiring, chest pains, coldness, anxiety [23-25], tiredness, weakness, indisposition, vomiting and epigastrical discomfort [26].

The most frequent symptoms in periods of AF reported by our group of patients included palpitations, reduced physical performance, palpitations when at rest, breathlessness when exerting oneself and anxiety. In an earlier report, the most pronounced symptoms were palpitations and anxiety as well as giddiness [24]. That females showed significantly higher frequencies of swollen legs, indisposition and anxiety than males has not previously been reported. These differences can possibly be accounted for since earlier studies have reported that attacks of AF in women last longer and cause higher heart-rates [27]. We could not, however, establish any significant differences in the lengths of attacks between men and women in our material.

Swollen legs can also be accounted for due to right-sided cardiac failure in some patients. That most patients experience definite symptoms following acute but transient attacks of AF can possibly depend on the increased activity of the sympathetic nervous system triggered by an attack of AF [28]. It is plausible to assume that the autonomic nervous system plays a considerable part in both the genesis of and the symptoms observed during a period of AF [24].

### Limitations

This material was taken at a hospital and is thus not representative of all patients with PAF. The symptoms of our patients are so far advanced that hospitalization or a visit to a hospital was required. Ongoing treatment can have modified patients' recollections of anamnestic factors. The material was not taken consecutively, but randomness was favoured by lack of a systemic inclusion mechanism.

Patients with hypertension are not excluded, even if subtle diastolic changes in the left ventricle and hence the left atrial performance could be caused by hypertension [29].

Although symptoms associated with the onset of PAF may be age related, the present material is too limited to allow exploration of this relation.

## Conclusions

Most of the patients in a group being treated at a hospital for PAF consider psychic stress to be the factor triggering their arrhythmia.

Red wine and spirits seems more prone to trigger attacks of AF than white wine.

The symptoms of PAF are many and occur frequently.

In women, PAF leads to significantly higher frequencies of swollen legs, indisposition and anxiety than in men.

## Competing interests

None.

## Authors' contributions

Author AH designed the investigation, collected all patient data, performed the statistical analysis and interpretation of the results, as well as the preparation of the manuscript.

Author BMH assisted with the statistical analysis and the preparation of the manuscript.

Author SBO supervised and designed the investigation as well as participated in the preparation of the manuscript.

All authors read and approved the final manuscript.

## Pre-publication history

The pre-publication history for this paper can be accessed here:



## Supplementary Material

Additional File 1 The complete questionnaire. The structured questionnaire with 58 questions covering arrhythmia-triggering factors, time at which the attack starts and symptoms during attack. Click here for file
