# Supplementary material for: Arrhythmia-provoking factors and symptoms at the onset of paroxysmal atrial fibrillation: A study based on interviews with 100 patients seeking hospital assistance
Source: BMC Cardiovasc Disord. 2004 Aug 3;4:13. doi: 10.1186/1471-2261-4-13 (PMC514544; doi:10.1186/1471-2261-4-13)
Supplement: Additional File 1 — The complete questionnaire. The structured questionnaire with 58 questions covering arrhythmia-triggering factors, time at which the attack starts and symptoms during attack. [file 1471-2261-4-13-S1.pdf]

FRÅGEFORMULÄR PAROXYSMALT FÖRMAKSFLIMMER

1. Har Ni släktingar som har eller har haft hjärtklappningsbesvär?

Föräldrar
Syskon
Barn
Andra släktingar

2. Har Ni tidigare haft hjärtinfarkt?
När? ____
Lokalisation ____________________________________

3. Har Ni tidigare haft hjärtsäcksinflammation?

4. Har Ni tidigare haft sköldkörtelsjukdom?

5. Har Ni sockersjuka (diabetes)?

6. Har Ni vid något tillfälle haft behandling för hypertoni?

7. Tidigare cerebral insult?

8. Har Ni någon neurologisk sjukdom?
Vilken? ____

9. Har Ni eller har Ni tidigare haft någon annan sjukdom?

10. När hade Ni hjärtklappning första gången?

11. Hur ofta har/hade Ni hjärtklappning?
Under tiden:____________________________
Utan medicinering

Färre än 1 gång per år
1-3 gånger per år
4-6 gånger per år
7-9 gånger per år
10-11 gånger per år
1-2 gånger per månad
3-4 gånger per månad
1-2 gånger per vecka
3-6 gånger per vecka
1 gång per dag
Fler än 1 gång per dag
Kan ej avgöra

12. Hur ofta har/hade Ni hjärtklappning?
Under tiden:____________________________
Med följande medicinering:

Färre än 1 gång per år
1-3 gånger per år
4-6 gånger per år
7-9 gånger per år
10-11 gånger per år
1-2 gånger per månad
3-4 gånger per månad
1-2 gånger per vecka
3-6 gånger per vecka
1 gång per dag
Fler än 1 gång per dag
Kan ej avgöra

13. Hur ofta har/hade Ni hjärtklappning?
Under tiden:___________________________
Med följande medicinering:

- Färre än 1 gång per år
- 1-3 gånger per år
- 4-6 gånger per år
- 7-9 gånger per år
- 10-11 gånger per år
- 1-2 gånger per månad
- 3-4 gånger per månad
- 1-2 gånger per vecka
- 3-6 gånger per vecka
- 1 gång per dag
- Fler än 1 gång per dag
- Kan ej avgöra

14. Hur ofta har Ni hjärtklappning?
Under tiden:___________________________
Med följande medicinering:

- Färre än 1 gång per år
- 1-3 gånger per år
- 4-6 gånger per år
- 7-9 gånger per år
- 10-11 gånger per år
- 1-2 gånger per månad
- 3-4 gånger per månad
- 1-2 gånger per vecka
- 3-6 gånger per vecka
- 1 gång per dag
- Fler än 1 gång per dag
- Kan ej avgöra

15. Hur länge varar i allmänhet varje hjärtklappningsepisod?

- Längre än 1 vecka
- 4-7 dygn
- 1-3 dygn
- 12-23 timmar
- 3-11 timmar
- 1-2 timmar
- 30-59 minuter
- Kortare än 15 minuter
- Kan ej avgöra

16. I vilka situationer får Ni hjärtklappning?

- Psykisk stress
- Fysisk ansträngning
- Vila
- Vaknar med det
- Kan ej avgöra

17. När på dygnet börjar hjärtklappningen?

- 00.00 – 03.00
- 03.01 – 06.00
- 06.01 – 09.00
- 09.01 – 12.00
- 12.01 – 15.00
- 15.01 – 18.00
- 18.01 – 21.00
- 21.01 – 23.59
- Inget säkert tidsintervall

18. Kan Ni själv bryta hjärtklappningsepisoden?
Om Ni svarat ja, på vilket sätt kan Ni bryta episoden? _________________________________
____

19. Hur många gånger har Ni blivit elkonverterad?

20. Symptom vid paroxysmalt förmaksflimmerepisod:

Förkänningar av något slag		Mycket	Måttligt	Lite	Ingen
Bröstsmärta		Mycket	Måttligt	Lite	Ingen
Yrsel		Mycket	Måttligt	Lite	Ingen
Svimning		Mycket	Måttligt	Lite	Ingen
Andfåddhet	i vila	Mycket	Måttligt	Lite	Ingen
Andfåddhet	vid ansträngning	Mycket	Måttligt	Lite	Ingen
Bensvullnad		Mycket	Måttligt	Lite	Ingen
Hjärtklappning	i vila	Mycket	Måttligt	Lite	Ingen
Hjärtklappning	vid ansträngning	Mycket	Måttligt	Lite	Ingen
Illamående		Mycket	Måttligt	Lite	Ingen
Kräkningar		Mycket	Måttligt	Lite	Ingen
Buksmärtor		Mycket	Måttligt	Lite	Ingen
Aptitlöshet		Mycket	Måttligt	Lite	Ingen
Ångest		Mycket	Måttligt	Lite	Ingen
Nedsatt fysisk prestationsförmåga		Mycket	Måttligt	Lite	Ingen
Ökat vattenkastningsbehov		Mycket	Måttligt	Lite	Ingen

Andra symptom: ____

____

21. På vilket sätt har Ni eventuella förkänningar? ____________________________________

____

22. Faktorer som kan utlösa episoder med paroxysmalt förmaksflimmer:

- Läkemedel — Vilka läkemedel________________________________

  ____
- Infektioner
- Trötthet
- Fysisk ansträngning
- Psykisk stress
- Födoämnen
  - Mandelmassa
  - Nötter
  - Choklad
  - Lök
  - Glass
  - Andra födoämnen
- Alkohol
  - Vitt vin
  - Rött vin
  - Starksprit
- Kaffe
- Nikotin
- Brandrök
- Andra utlösande faktorer____

23. Hur mycket röker Ni?

Har aldrig rökt
Har tidigare rökt men slutat
Slutade för _______ år sedan
Hade då rökt under _______ år
Röker 1-29 cigaretter per månad
Röker 1-3 cigaretter per dag
Röker 4-6 cigaretter per dag
Röker 7-15 cigaretter per dag
Röker 16-20 cigaretter per dag
Röker mer än 20 cigaretter per dag
Röker pipa

24. Snusar Ni?
Hur mycket?

25. Hur mycket kaffe dricker Ni?

Dricker inte kaffe
Dricker mindre än 1 kopp per dag
Dricker 1-2 koppar per dag
Dricker 3-5 koppar per dag
Dricker 6-10 koppar per dag
Dricker mer än 10 koppar per dag

26. Hur mycket alkohol har Ni druckit senaste månaden?

Har inte druckit några alkoholhaltiga drycker

Antal lättöl ______
Antal starköl _____
Mängd vin ______
Mängd starksprit ______

LIVSKVALITET

27. Påverkar episoderna av paroxysmalt förmaksflimmer Din livsföring?
Mycket Måttligt Lite Inget

28. På vilket sätt påverkar episoderna Dig? ____

29. Vad är det som Du skulle vilja göra men som Du inte kan eller orkar? ___________________________
____

30. Påverkar oron mellan förmaksflimmerepisoderna Din livsföring?
Mycket Måttligt Lite Inget Har ingen oro

31. Påverkar episoderna med paroxysmalt förmaksflimmer Dig i Ditt yrke?
Mycket Måttligt Lite Inget

32. Påverkar episoderna med paroxysmalt förmaksflimmer även Dina anhöriga?
Mycket Måttligt Lite Inget
På vilket sätt? ____

33. Antiarytmika som provats:

Digitalis	effekt:	God	Måttlig	Ingen	Biverkningar
B-blockerare	effekt:	God	Måttlig	Ingen	Biverkningar
Flekainid	effekt:	God	Måttlig	Ingen	Biverkningar
Kinidin	effekt:	God	Måttlig	Ingen	Biverkningar
Verapamil	effekt:	God	Måttlig	Ingen	Biverkningar
Disopyramid	effekt:	God	Måttlig	Ingen	Biverkningar
Sotalol	effekt:	God	Måttlig	Ingen	Biverkningar
Amiodaron	effekt:	God	Måttlig	Ingen	Biverkningar
________________	effekt:	God	Måttlig	Ingen	Biverkningar

34. Aktuell medicinering:

35. Diagnosen paroxysmalt förmaksflimmer ställd hur och när?
